# Intracranial pressure guided management of patients with Chiari malformations presenting with headache: a paradigm shift?

**DOI:** 10.1186/2045-8118-12-S1-O29

**Published:** 2015-09-18

**Authors:** Edward W Dyson, Aswin Chari, Andrew R Stevens, Simon D Thompson, Claudia Craven, Patricia Haylock-Vize, Samir A Matloob, Syed N Shah, Huan Wee Chan, Neekhil A Patel, Tarek Mostafa, Jinendra Ekanayake, Ahmed K Toma, Lewis W Thorne, Laurence D Watkins

**Affiliations:** 1 Victor Horsley Department of Neurosurgery, National Hospital for Neurology & Neurosurgery, Queen Square, London, UK

## Introduction

Chiari malformation (CM) describes cerebellar tonsillar descent below the level of the foramen magnum. It is commonly associated with syringomyelia and often presents with headache (1). The conventional surgical treatment for symptomatic patients is foramen magnum decompression (FMD) (2) which carries a significant burden of operative morbidity (3). Altered cerebrospinal fluid (CSF) dynamics have been demonstrated in CM patients and CSF diversion has been used as an alternative treatment modality. Patients with chronic headache and radiological evidence of CM represent a therapeutic challenge. In our unit, these are primarily investigated with intracranial pressure (ICP) monitoring aiming to detect objective evaluation of CSF dynamics prior to surgical intervention.

## Methods

In this single centre, retrospective study, CM patients presenting with headaches were extracted from our departmental ICP monitoring database. Patients with an existing CSF diversion shunt or a previous foramen magnum decompression were excluded. ICP monitoring results were analysed with emphasis on median intracranial pressure (mICP) and median pulse amplitude (mPA). Clinical records were reviewed for clinical presentation, surgical management and outcome.

## Results

16 patients with CM and ICP monitoring were identified. 7 had associated syringomyelia. The mean mICP across the group was 2.97 ± 3.13 mmHg. Mean mPA was 5.23 ± 1.27 mmHg (normal value ≤ 4 mmHg). All patients had mICP < 10 mmHg. 2 patients had mICP < 0 mmHg. 14 out of 16 patients had abnormal pulsatility (mPA > 4 mmHg). 6 patients were treated with primary ventriculoperitoneal shunt (VPS) insertion, 3 underwent FMD, 1 was treated medically with acetazolamide and 5 were managed conservatively. There were no significant surgical complications in either the VPS or the FMD group. At 2 month follow-up, all patients in the VPS group experienced symptomatic improvement. 2 patients in the FMD group experienced symptomatic improvement and one was unchanged.

## Conclusions

The majority of patients with symptomatic untreated Chiari malformation have increased ICP pulsatility (and by deduction, impaired compliance) but a “normal” overall mICP. Raw ICP values are not sensitive in identifying abnormalities of compliance in this patient group. A small group of patients may develop tonsilar descent due to relatively low ICP.

VPS insertion may be a safe, effective alternative to FMD for patients with symptomatic CM, even in the absence of hydrocephalus.

We propose routine intracranial pressure monitoring in CM patients with headache and, if indicated, cranial CSF diversion as first line management.
